# UNICORN for Valve-in-Valve Transcatheter Aortic Valve Replacement: UNICORN Hong Kong Registry

**DOI:** 10.1016/j.jacasi.2025.10.011

**Published:** 2025-11-17

**Authors:** Chun-Ka Wong, Kwong-Yue Eric Chan, Daniel Tai-Leung Chan, Ho-On Alston Conrad Chiu, Ka-Chun Un, Shu-Yue Sze, Frankie Chor-Cheung Tam, Cheuk-Wing Lee, Max Kwun-Hung Wong, Ming Lau, Pik-Ki Law, Audrey Tsznam Ko, Joyce Shek, Tsun-Ho Lam, Yee-Tat Lee, Martin Kin-Lam Cheng, Minqing Lin, Yui-Ming Lam, Gilbert H.L. Tang, Simon Cheung-Chi Lam

**Affiliations:** a Cardiology Division, Department of Medicine, School of Clinical Medicine, Li Ka Shing Faculty of Medicine, The University of Hong Kong, Hong Kong SAR, China; b Cardiology Division, Department of Medicine, Queen Mary Hospital, Hong Kong SAR, China; c Cardiac Medical Unit, Grantham Hospital, Hong Kong SAR, China; d Department of Surgery, School of Clinical Medicine, Li Ka Shing Faculty of Medicine, The University of Hong Kong, Hong Kong SAR, China; e Department of Cardiothoracic Surgery, Queen Mary Hospital, Hong Kong SAR, China; f Department of Cardiovascular Surgery, Mount Sinai Health System, New York, New York, USA

**Keywords:** transcatheter aortic valve replacement, transcatheter electrosurgery, valve-in-valve transcatheter aortic valve replacement, UNICORN technique

## Abstract

**Background:**

Undermining Iatrogenic Coronary Obstruction With Radiofrequency Needle (UNICORN) was developed to mitigate coronary obstruction risk during valve-in-valve transcatheter aortic valve replacement (ViV-TAVR) with balloon-expandable valves (BEVs). A modified UNICORN with recrossing technique was subsequently described for self-expanding valves (SEVs).

**Objectives:**

This study aimed to assess early outcomes of the UNICORN technique.

**Methods:**

The UNICORN Hong Kong Registry was a single-center, retrospective observational study at Queen Mary Hospital, Hong Kong, including consecutive patients undergoing ViV-TAVR between July 2024 and August 2025. Patients were treated with either UNICORN using BEV or UNICORN with recrossing using SEV. The primary endpoint was procedural success, defined as successful leaflet traversal, intended leaflet laceration, and transcatheter heart valve implantation without procedural mortality, coronary obstruction, or emergency surgery. Secondary outcomes included 30-day safety events per Valve Academic Research Consortium-3.

**Results:**

Seventeen patients (41.2% male; 78.3 ± 6.61 years) underwent ViV-TAVR: 8 with BEV and 9 with SEV using the recrossing technique. All SEV patients had prior surgical valves, whereas in the BEV group 50.0% had surgical valves and 50.0% had transcatheter valves. Coronary ostium at risk was left in 58.8%, right in 29.4%, and bilateral in 11.8%. Median coronary height was 7.80 [7.00-8.50] mm and mean virtual transcatheter heart valve to coronary distance was 4.56 ± 1.48 mm. One patient required bileaflet modification with concomitant UNICORN and Bioprosthetic or Native Aortic Scallop Intentional Laceration to Prevent Iatrogenic Coronary Artery Obstruction. Procedural success was achieved in all patients with no coronary obstruction. At 30 days, 1 patient (5.88%) required pacemaker implantation.

**Conclusions:**

The UNICORN technique was feasible for preventing coronary obstruction during ViV-TAVR and warrants further evaluation in larger trials.

Undermining Iatrogenic Coronary Obstruction With Radiofrequency Needle (UNICORN) was first described in 2022 as a technique to mitigate the risk of coronary obstruction during valve-in-valve transcatheter aortic valve replacement (ViV-TAVR).^1^^,^^2^ The procedure begins with traversal of the target leaflet base using a radiofrequency wire. A noncompliant coronary balloon followed by a peripheral balloon is used for serial dilatation of the leaflet without laceration. A balloon-expandable valve (BEV) is then advanced through the fenestration for intraleaflet deployment. Because leaflet laceration and valve implantation occur simultaneously, this approach avoids hemodynamic compromise from acute aortic regurgitation. In addition, continuous contact between the BEV and the annular surface adjacent to the coronary ostium creates a barrier that prevents leaflet prolapse into the coronary artery.

Interests have grown in adapting UNICORN for self-expanding valves (SEVs).^3^^,^^4^ However, SEVs do not exert sufficient radial force to lacerate the leaflet when deployed within the fenestration. As a result, many operators have used oversized balloons to achieve leaflet laceration before SEV implantation, which introduces a period of aortic regurgitation between the 2 steps. Recently, we reported the first-in-human experience of the UNICORN recrossing technique, which enables a seamless SEV procedure similar to the original BEV approach while minimizing the duration of aortic regurgitation.^5^

Real-world data on UNICORN with BEV and SEV with recrossing technique remain limited. To address this gap, the UNICORN Hong Kong Registry was established to evaluate the safety and efficacy of this approach in clinical practice. The registry includes ViV-TAVR procedures performed with either BEV using the original UNICORN technique or SEV using the recrossing technique.

## Methods

### Study design

The UNICORN Registry is a single-center, single-arm, retrospective observational study conducted at Queen Mary Hospital, Hong Kong. It included consecutive patients who underwent ViV-TAVR between July 2024 and August 2025 using UNICORN with BEV or UNICORN with SEV and recrossing technique using newer-generation transcatheter heart valves (THVs). Safety endpoints were assessed according to Valve Academic Research Consortium-3 (VARC-3) definitions.^6^

### Ethics statement

Ethical approval was obtained from the institutional review boards of the University of Hong Kong and the Hospital Authority Hong Kong West Cluster (HKU/HA HKW IRB; UW 12-177). The study was conducted in accordance with the Declaration of Helsinki, and reporting followed the STrengthening the Reporting of OBservational studies in Epidemiology (STROBE) checklist.^7^

### Study population

Consecutive adult patients aged ≥18 years old treated between July 2024 and August 2025 at Queen Mary Hospital, Hong Kong, with either UNICORN with BEV or UNICORN with SEV and recrossing technique for ViV-TAVR were included. Patients with the newest generation THV SAPIEN 3 Ultra RESILIA (Edwards Lifesciences) and Evolut FX+ (Medtronic) were included. Patients were excluded if they received TAVR via alternative vascular access, if SEV was used without the recrossing technique, or if older-generation THVs were implanted.

### Preoperative computed tomography assessment

Preoperative computed tomography (CT) analysis was performed using 3mensio version 10.7 (Pie Medical Imaging). Virtual transcatheter heart valve to coronary distance (VTC) was defined as the shortest distance between the outer edge of the virtual THV and the coronary ostium.^8^^,^^9^ Coronary height was measured as the distance between the annular plane and the lower edge of the coronary ostium. In addition, multiple other risk factors were considered to determine coronary obstruction risk, including the type and design of the previously implanted valve, such as those with externally mounted leaflets, as well as commissural alignment. Optimal projection angles for leaflet traversal were determined from CT reconstructions. The side view was obtained when the markers of the 2 nontarget coronary cusps overlapped. The en face view was obtained by rotating the model until the target coronary ostium was centered within the target leaflet and positioned midway between the commissures.

The choice between BEV and SEV was based on both anatomical and procedural considerations. SEV offers advantages such as better hemodynamics with supra-annular designs and more reliable commissural alignment adjustments in newer-generation devices. However, SEV may also present challenges, including a higher risk of sinus sequestration and technically difficult rescue chimney stenting in patients with small aortic root anatomy and high coronary obstruction risk, as well as limited opening force and radial strength for deployment within fenestration without balloon-assisted techniques.

### Leaflet modification strategy

Our approach for selecting leaflet modification strategy depends on the anatomical and other patient-specific considerations. For native TAVR cases, only BASILICA is considered. When an isolated coronary ostium is at risk with good commissural alignment, both BASILICA and UNICORN are viable options; in right coronary cusp (RCC) cases, UNICORN may be technically less challenging. If the at-risk coronary ostium has suboptimal commissural alignment, UNICORN may be preferred. Patients with bilateral coronary ostia at risk present greater procedural complexity compared with those at unilateral risk. A case-by-case evaluation is therefore essential, considering anatomical factors, patient condition including hemodynamic status and comorbidities, and technical aspects such as the complexity of performing bileaflet modification and the potential difficulty of bailout stenting if required. In these situations, the optimal strategy is selected individually, which may include Doppio BASILICA, concomitant UNICORN-BASILICA, or snorkel and chimney stenting.

### UNICORN using BEV

**Figure 1:**
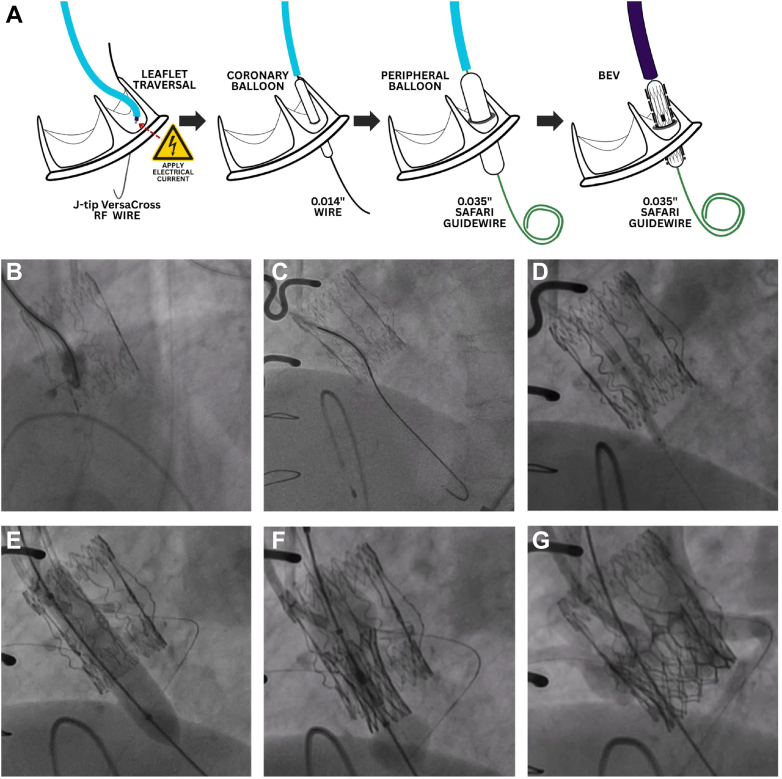
UNICORN With BEV (A) Illustration showing key steps of Undermining Iatrogenic Coronary Obstruction With Radiofrequency Needle (UNICORN) with balloon-expandable valve (BEV). (B) Contrast injection in side view confirmed the leaflet traversal site. (C) Leaflet traversal was performed with a J-tip VersaCross RF Wire. (D) The first dilatation was carried out with a coronary balloon. (E) The second dilatation was performed with a peripheral balloon. The leaflet was not lacerated at this stage. (F) The BEV was inserted and deployed inside the fenestration. (G) Patent coronary flow was demonstrated after BEV implantation.

Leaflet traversal is performed using a telescoping system comprising a J-tip VersaCross RF Wire (Boston Scientific), a 135-cm NaviCross (Terumo), and a 7-F guiding catheter (Figure 1A). The guiding catheters are typically AL-1 or AL-2 for the left coronary cusp (LCC) and AL-1, AL-2, or Multipurpose for the RCC. The telescoping system is directed toward the base of the target leaflet under transesophageal echocardiography (TEE) and fluoroscopic guidance. Both the side view and en face view identified during preoperative CT assessment are used for orientation. The system is angled toward the center rather than outward. Precise localization of the traversal site is confirmed by contrast injection into the target cusp (Figure 1B). The target leaflet is traversed with a radiofrequency impulse (Figure 1C). After advancing the NaviCross into the left ventricle, the guidewire is exchanged for a 300-cm coronary wire. Initial dilatation of the fenestration is performed with a 3.0-mm coronary balloon (Figure 1D). The guidewire is then exchanged for a pre-shaped stiff wire such as a SAFARI^2^ guidewire (Boston Scientific). A second dilatation is performed with a 10- to 12-mm peripheral balloon (Figure 1E). At this stage, the target leaflet is ideally not lacerated. The BEV is subsequently positioned inside the fenestration of the target leaflet (Figure 1F). Inflation of the BEV simultaneously opens the target leaflet and implants the THV (Figure 1G).

When crossing the fenestration with the BEV is challenging, a bailout recrossing technique is used. A second guidewire, such as a straight-tip EMERALD (Cordis), is introduced via contralateral 7F vascular access in a 5F diagnostic catheter to recross the fenestration. This guidewire is then exchanged for an Amplatz Extra Stiff guidewire. A larger peripheral balloon is subsequently delivered to further dilate the fenestration through the second guidewire. During this process, the BEV remains parked in the aorta until definitive implantation.

### UNICORN with SEV and recrossing technique

**Figure 2:**
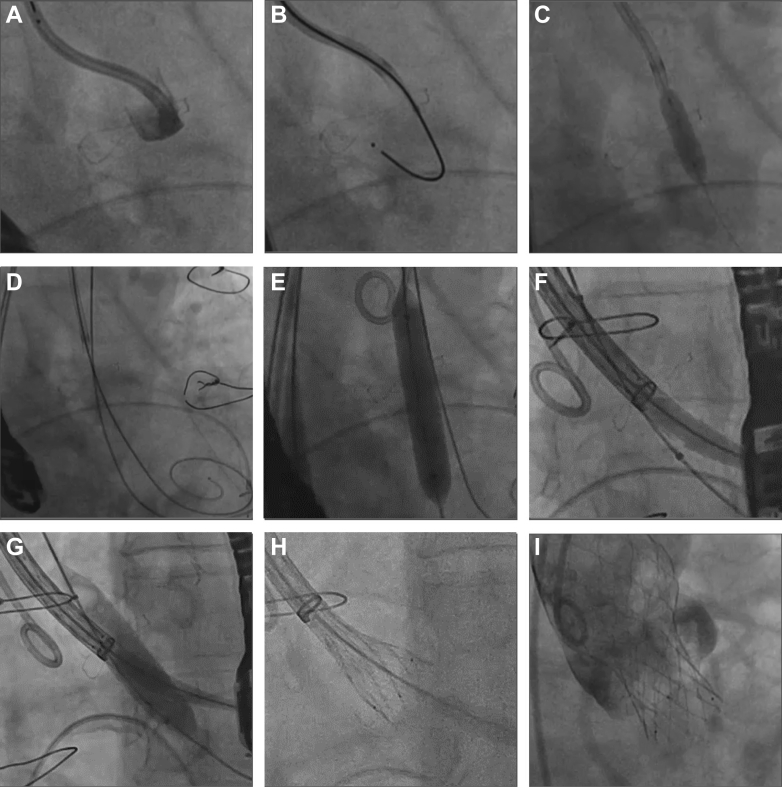
UNICORN With SEV and Recrossing Technique (A) Leaflet traversal site confirmed with contast injection. (B) Leaflet traversal using J-tip VersaCross RF Wire. (C) First dilatation with coronary balloon. (D) Second guidewire was inserted via contralateral access to recross the fenestration. In total, 2 guidewires were positioned across the fenestration. (E) A self-expanding valve (SEV) was delivered via the main vascular access and first guidewire to the descending aorta and adjusted to facilitate commissural alignment. An 8-mm peripheral balloon was delivered via contralateral access and the second wire to dilate the fenestration without lacerating it. (F) Side-by-side approach with SEV and 10-mm peripheral balloon positioned in the fenestration. (G) Leaflet laceration with 10-mm peripheral balloon. (H) SEV deployed immediately as the peripheral balloon deflated. (I) Patent coronary flow. Abbreviation as in Figure 1.

Using a telescoping system to traverse the target leaflet with a J-tip VersaCross RF Wire (Figures 2A and 2B), fenestration dilatation is performed with a 5.0-mm noncompliant coronary balloon (Figure 2C). The first guidewire is then exchanged for a pre-shaped stiff wire such as SAFARI.^2^ A second guidewire within a 5-F diagnostic catheter is introduced through contralateral 7-F vascular access, for example a straight-tip EMERALD (Cordis), to recross the fenestration. This second guidewire is then exchanged for an Amplatz Extra Stiff guidewire, resulting in 2 guidewires positioned across the fenestration (Figure 2D). Through the primary access and first guidewire, the Evolut FX+ system is advanced to the descending aorta and adjusted so that the delivery system head marker aligns with the outer curvature under left anterior oblique projection to facilitate commissural alignment. The peripheral balloon is delivered over the second guidewire. From this point, 2 variations of the recrossing technique are applied.

In the sequential approach, if the target leaflet is lacerated after peripheral balloon inflation, then the SEV, already parked in the descending aorta, is immediately advanced to the aortic valve level for deployment.

In the side-by-side approach, initial fenestration dilatation with an 8-mm peripheral balloon can leave the leaflet intact (Figure 2E). A larger 10- to 12-mm peripheral balloon and the SEV capsule are then positioned within the fenestration in parallel (Figure 2F). Under controlled pacing to stabilize positions, the balloon is inflated to lacerate the leaflet (Figure 2G) and the SEV is deployed immediately as the balloon deflates (Figures 2H and 2I).

Both strategies minimize the duration of aortic regurgitation, which is particularly advantageous in patients with unstable hemodynamics.

### Bileaflet modification with BASILICA and UNICORN

**Figure 3:**
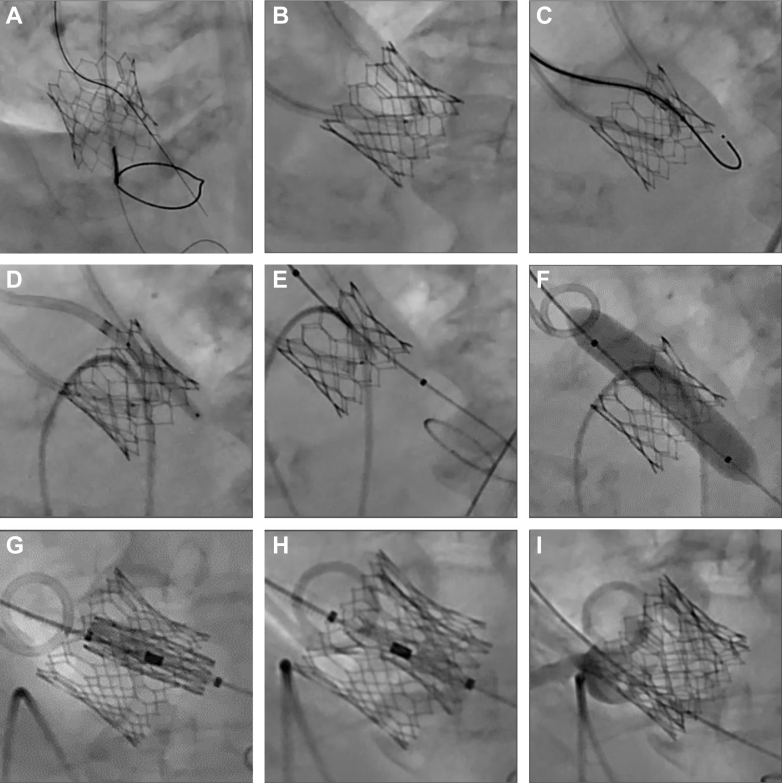
Bileaflet Modification with UNICORN and BASILICA (A) After traversing the first target leaflet, Astato XS 20 guidewire was snared in the left ventricular outflow tract. (B) A “flying V” was created by stripping insulation and bending the Astato wire near the converter tip. (C) Second target leaflet was traversed using J-tip VersaCross RF Wire. (D) Second target leaflet was dilated with coronary balloon. (E) Peripheral balloon was positioned inside the fenestration of the second target leaflet without inflation. The balloon insulated the left ventricular wire in the second target leaflet during Bioprosthetic or Native Aortic Scallop Intentional Laceration to Prevent Iatrogenic Coronary Artery Obstruction (BASILICA) of the first target leaflet. (F) After completion of BASILICA laceration of the first target leaflet, the peripheral balloon in the second target leaflet dilated the fenestration. (G and H) BEV was inserted into the fenestration and deployed. (I) Patent coronary flow. Abbreviations as in Figure 1.

For the first target leaflet, a catheter is advanced across the aortic valve into the left ventricular outflow tract, where a snare is deployed below the valve. A 0.018-inch safety wire is placed in the left ventricle. An aortic catheter carrying a 0.014-inch Astato XS 20 guidewire (Asahi Intecc Medical) inside a 0.035-inch microcatheter is then directed to the base of the leaflet. Electrocautery is applied to traverse the valve, and the guidewire is captured by the snare in the outflow tract (Figure 3A). To prepare for leaflet laceration, a “flying V” is fashioned by stripping insulation and bending the Astato wire near the microcatheter’s tip (Figure 3B). This configuration is repositioned at the leaflet and secured with torque devices.

**Central Illustration:**
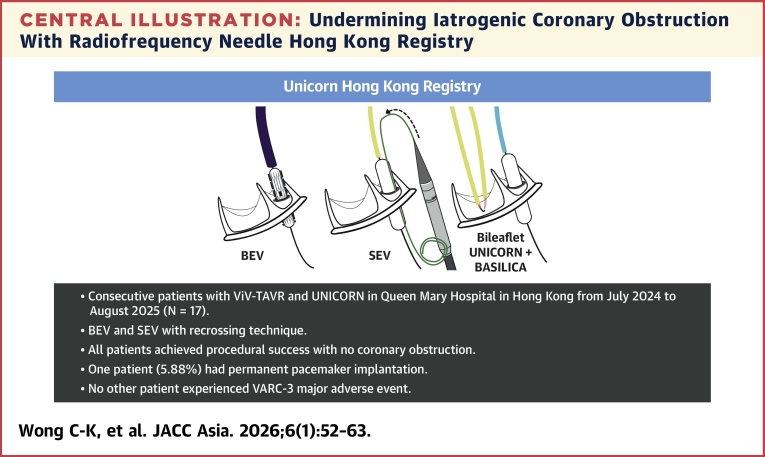
Undermining Iatrogenic Coronary Obstruction With Radiofrequency Needle Hong Kong Registry In this single-center, retrospective observational study involving consecutive patients who receive valve-in-valve transcatheter aortic valve replacement (ViV-TAVR) with high coronary obstruction risk between July 2024 and August 2025, Undermining Iatrogenic Coronary Obstruction With Radiofrequency Needle (UNICORN) using balloon-expandable valve (BEV) and self-expanding valve (SEV) using a novel recrossing technique was found to be safe and effective. VARC-3 = Valve Academic Research Consortium-3.

For the second target leaflet, a telescoping system consisting of a 7-F guiding catheter, a 135-cm NaviCross, and a 0.035-inch J-tip VersaCross RF Wire is positioned at the leaflet base. After radiofrequency traversal (Figure 3C), the guidewire is exchanged for a 300-cm coronary wire. The fenestration is first dilated with a 3.0-mm noncompliant coronary balloon (Figure 3D). The coronary wire is then exchanged for a pre-shaped stiff wire, and a 10- to 12-mm peripheral balloon is advanced into the fenestration. This peripheral balloon remains uninflated within the fenestration to electrically insulate the left ventricular wire across the second target leaflet during BASILICA of the first leaflet (Figure 3E).

BASILICA is then performed on the first target leaflet. During laceration, 5% dextrose is infused through both catheters. Gentle counter-traction is maintained to achieve a clean split without mechanical tearing. The procedure then continues with UNICORN of the second leaflet. After dilatation with the peripheral balloon, the fenestration enlarges while the leaflet remains intact (Figure 3F). A BEV is then advanced into the fenestration and deployed within the leaflet (Figures 3G to 3I).

### Echocardiographic assessment

Transthoracic echocardiography and TEE were performed by cardiologists with expertise in imaging for structural heart interventions, using EPIQ CVxi (Philips) and Vivid E95 (GE HealthCare). Aortic stenosis and regurgitation severity were classified into 4 grades according to established echocardiographic criteria.^10^ Both peak and mean aortic valve pressure gradients were recorded. Left ventricular size and function were evaluated by end-diastolic volumes and by left ventricular ejection fraction.

### Study outcomes

The primary endpoint was procedural success, which was defined as successful leaflet traversal, laceration of the intended leaflet(s), successful implantation of the THV in the absence of procedural mortality, absence of coronary artery obstruction, and freedom from emergency cardiac surgery or intervention, measured at exit from the catheterization laboratory.^11^

Secondary endpoints included incidence of major adverse events at 30 days, as defined by the VARC-3 criteria.^6^ This composite endpoint encompassed all-cause mortality, stroke, major vascular complication, life-threatening bleeding, major cardiac structure complication, acute kidney injury, coronary artery obstruction requiring intervention, myocardial infarction, any reintervention for valve dysfunction, and conduction disturbances requiring permanent pacemaker implantation.

### Statistical analysis

Normality of continuous variable was evaluated using Shapiro-Wilk test. Continuous variables were expressed as mean ± SD or median [IQR] depending on normality. Pairwise comparison for continuous variable was performed using Student's *t*-tests, Mann-Whitney *U* test, and Wilcoxon signed-rank test. Categorical variables were summarized as frequencies and percentages. Pairwise comparison between categorical variables was performed using chi-square test or Fisher’s exact test. *P* values <0.05 were considered statistically significant. Analyses were performed using python v3.11.7, pandas v2.0.2, numpy v1.24.3, and scipy v1.10.1.

## Results

### Baseline characteristics

Between July 2024 and August 2025, 17 patients (41.2% male; mean age 78.3 ± 6.61 years) underwent transfemoral ViV-TAVR using the UNICORN technique. Eight patients received a SAPIEN 3 Ultra RESILIA BEV, and 9 received an Evolut FX+ SEV with the recrossing technique (Table 1). The most common medical conditions were coronary artery disease (64.7%), chronic kidney disease (52.9%), hypertension (41.2%), diabetes mellitus (29.4%), and atrial fibrillation (29.4%). Overall, the cohort had high surgical risk with a mean EuroSCORE II of 13.2% ± 7.07% and a median Society of Thoracic Surgeons score of 10.3% [9.06%, 21.1%].^12^^,^^13^

**Table 1:** Baseline Characteristics

	Overall (N = 17)	BEV (n = 8)	SEV with Recrossing (n = 9)	*P* Value
Male	7 (41.2)	4 (50.0)	3 (33.3)	0.637
Age, y	78.3 ± 6.61	81.8 ± 7.36	75.3 ± 4.21	0.0501
Hypertension	7 (41.2)	4 (50.0)	3 (33.3)	0.637
Diabetes mellitus	5 (29.4)	2 (25.0)	3 (33.3)	1.00
Chronic kidney disease^a^	9 (52.9)	4 (50.0)	5 (55.6)	1.00
Chronic obstructive pulmonary disease	0 (0)	0 (0)	0 (0)	1.00
Coronary artery disease	11 (64.7)	6 (75.0)	5 (55.6)	0.62
Myocardial infarction	2 (11.8)	0 (0)	2 (22.2)	0.471
Percutaneous coronary intervention	0 (0)	0 (0)	0 (0)	1.00
Coronary artery bypass graft surgery	2 (11.8)	0 (0)	2 (22.2)	0.471
Cardiac implantable electronic device	2 (11.8)	1 (12.5)	1 (11.1)	1.00
Ischemic stroke	1 (5.88)	0 (0)	1 (11.1)	1.00
Hemorrhagic stroke	1 (5.88)	1 (12.5)	0 (0)	0.471
Atrial fibrillation	5 (29.4)	2 (25.0)	3 (33.3)	1.00
EuroSCORE II	13.2 ± 7.07	14.2 ± 8.70	12.3 ± 5.64	0.601
STS score	10.3 [9.06, 21.1]	16.6 [9.68, 24.9]	10.2 [9.06, 11.7]	0.277

Values are n (%) unless otherwise indicated.

BEV = balloon-expandable valve; SEV = self-expanding valve; STS = Society of Thoracic Surgeons.

a Chronic kidney disease stage 3 or above with estimated glomerular filtration rate (eGFR) <60 mL/min/1.73 m^2^.

## Preoperative assessment

The indication for ViV-TAVR was severe aortic stenosis in 12 patients (70.6%), mixed severe aortic stenosis and regurgitation in 3 patients (17.6%), and severe aortic regurgitation in 2 patients (11.8%). All patients in the SEV with recrossing group had existing surgical valves in situ. In the BEV group, 50.0% had existing surgical valves and 50.0% had older-generation transcatheter valves in situ. The new THVs implanted were relatively small in this exclusively Chinese cohort. In the SEV with recrossing group, 88.9% received an Evolut FX+ 23 mm and 11.1% received a 26 mm. In the BEV group, 50.0% received a SAPIEN 3 Ultra RESILIA 20 mm, 37.5% received a 23 mm, and 12.5% received a 26 mm. Coronary ostium at risk was left coronary cusp (LCC) in 58.8%, RCC in 29.4%, and bilateral in 11.8%. The median coronary height of the UNICORN target was 7.80 [7.00, 8.50] mm and the mean VTC was 4.56 ± 1.48 mm (Table 2).

**Table 2:** Tools Used for Individual Patients

	Existing Valve (Size)	New Valve (Size)	UNICORN Target Leaflet	Coronary Height, mm^a^	VTC, mm^a^	Leaflet Traversal Tool	First Balloon, mm	Second Balloon, mm
1	Perceval S Small (23)	S3UR (20)	RCC	3.8 (4.3)	1.3 (5.0)	VersaCross J-tip	NC 4.0	10.0
2	Evolut Pro (29)	S3UR (23)	LCC	11.2^b^	5.7^b^	VersaCross J-tip	NC 3.0	12.0
3	Trifecta (21)	S3UR (20)	RCC	4.7	7.2	VersaCross J-tip	NC 3.0	10.0
4	Sapien XT (23)	S3UR (20)	LCC	9	3.6	VersaCross J-tip	NC 3.5	8.0
5	Trifecta (19)	Evolut FX+ (23)	LCC	8	7.2	VersaCross J-tip	NC 5.0	—
6	Trifecta (19)	Evolut FX+ (23)	LCC	7.6	4.5	VersaCross J-tip	NC 5.0	8.0
7	Mitroflow (23)	S3UR (23)	LCC	7.3	6	VersaCross J-tip	NC 3.0	10.0^c^
8	Trifecta (19)	Evolut FX+ (23)	RCC	7.9	4	VersaCross J-tip	NC 5.0	8.0
9	Trifecta (23)	Evolut FX+ (26)	LCC	7.4	5.2	Astato XS 20 GW	NC 5.0	10.0
10	Trifecta (18)	Evolut FX+ (23)	LCC	8	4	VersaCross J-tip	NC 5.0	8.0
11	Epic (19)	Evolut FX+ (23)	RCC	6.4	4.5	VersaCross J-tip	NC 5.0	8.0
12	Magna Ease (21)	Evolut FX+ (23)	LCC	11.9	2.8	VersaCross J-tip	NC 5.0	8.0
13	Evolut Pro (29)	S3UR (23)	LCC	7	4.9	VersaCross J-tip	NC 3.0	10.0
14	Magna Ease (19)	Evolut FX+ (23)	RCC	8.9	4.2	VersaCross J-tip	NC 3.0	8.0
15	Sapien 3 (23)	S3UR (20)	LCC^d^	7.8 (8.0)	3.3 (3.6)	VersaCross J-tip	NC 3.0	10.0
16	Trifecta (25)	S3UR (26)	LCC	6.5	4.9	VersaCross J-tip	NC 3.0	12.0
17	Trifecta (21)	Evolut FX+ (23)	LCC	8.5	4.2	VersaCross J-tip	NC 5.0	8.0

BASILICA = Bioprosthetic or Native Aortic Scallop Intentional Laceration to Prevent Iatrogenic Coronary Artery Obstruction; LCC = left coronary cusp; NC = noncompliant; RCC = right coronary cusp; S3UR = SAPIEN 3 Ultra RESILIA; SC = semi-compliant; UNICORN = Undermining Iatrogenic Coronary Obstruction With Radiofrequency Needle; other abbreviation as in Table 1.

a Coronary height and virtual transcatheter heart valve to coronary distance of UNICORN target leaflet. If the other coronary ostium is also at risk of obstruction, measurement numbers are presented in parenthesis.

b Owing to advanced chronic kidney disease, no preoperative contrast-enhanced computed tomography was performed for this patient. Transesophageal echocardiography and 3-dimensional intracardiac echocardiography was used for evaluation.

c Initially used 8/40 mm for second dilatation. Fenestration recrossed and further dilated with 10/40 mm to facilitate crossing of BEV.

d Case of bileaflet modification with concomitant BASILICA performed to RCC.

### UNICORN procedure

In the BEV group, 7 patients underwent unilateral UNICORN and 1 patient underwent bileaflet modification with concomitant UNICORN and BASILICA. In the SEV with recrossing group, all 9 patients underwent unilateral UNICORN using the recrossing technique. All procedures were performed under general anesthesia with TEE guidance. One patient with advanced renal failure did not undergo preoperative contrast-enhanced CT. Instead, a combination of TEE and 3-dimensional intracardiac echocardiography was used to assess the risk of coronary obstruction and to guide the procedure. The SENTINEL Cerebral Protection System (Boston Scientific) was used in all cases. Leaflet traversal was successful in all patients, using a J-tip VersaCross RF Wire in 94.1% and an Astato XS 20 GW in 5.88%. The most common coronary balloon size for the first dilatation was 3.0 mm (75.0%) in the BEV group and 5.0 mm (88.9%) in the SEV with recrossing group. The most common peripheral balloon size for the second dilatation was 10.0 mm (62.5%) in the BEV group and 8.0 mm (87.5%) in the SEV with recrossing group. Procedural success was achieved in all patients with successful THV implantation and no coronary obstruction.

Patient 15 had bilateral coronary ostia at risk and required bileaflet modification with UNICORN to the LCC and BASILICA to the RCC. Step-by-step procedural details are provided in the Methods section (Figure 3, Table 2). For patient 7, an 8-mm peripheral balloon was initially used to dilate a Mitroflow prosthesis. However, there was difficulty in crossing the BEV. Bailout recrossing technique was then employed, with a second guidewire recrossing the fenestration through contralateral vascular access. A 10-mm balloon was subsequently used to further dilate the fenestration, which facilitated successful BEV crossing.

### Clinical outcome

At 30 days, 1 patient (5.88%) experienced a VARC-3 major adverse event, which was permanent pacemaker implantation for atrioventricular block. Prosthetic valve fracture was performed in that patient. No other VARC-3 adverse events were observed (Table 3). Functional status improved from preoperative NYHA functional class II in 47.1%, class III in 23.5%, and class IV in 29.4% to postoperative class I in 94.1% and class II in 5.9% (*P* < 0.001∗). (∗ indicates p value <0.05).

**Table 3:** Major Adverse Events at 30 Days (N = 17)

Major adverse events	1 (5.88)^a^
Coronary obstruction/myocardial infarction	0 (0)
Cardiac complication	0 (0)
Major vascular complication	0 (0)
Life-threatening bleeding	0 (0)
Ischemic stroke	0 (0)
Hemorrhagic stroke	0 (0)
Acute kidney injury	0 (0)
Emergent cardiac surgery	0 (0)
New permanent pacemaker	1 (5.88)^a^
All-cause mortality	0 (0)

Values are n (%).

a Prosthetic valve fracture was performed for this patient.

### Echocardiography

Peak and mean aortic valve pressure gradients decreased from 74.0 ± 22.2 mm Hg and 42.4 ± 13.9 mm Hg to 20.6 ± 7.19 mm Hg and 10.7 ± 4.00 mm Hg, respectively (*P* < 0.001∗). The proportion of patients with moderate to severe aortic regurgitation decreased from 50.0% to 0% (*P* = 0.00298∗). The proportion of patients with moderate to severe mitral regurgitation decreased from 50.0% to 6.25% (*P* < 0.001∗). There was no significant change in left ventricular ejection fraction (57.8% ± 8.36% vs 59.6% ± 9.33%, *P* = 0.399), left ventricular end-diastolic diameter (4.47 ± 0.805 cm vs 4.23 ± 0.75 cm, *P* = 0.0973), or the proportion of patients with moderate to severe tricuspid regurgitation (43.8% vs 12.5%, *P* = 0.0339).

## Discussion

After our initial description of the UNICORN technique,^1^ we report here a retrospective observational registry of patients who undergo ViV-TAVR using either UNICORN with BEV or UNICORN with SEV and the recrossing technique. The UNICORN Hong Kong Registry demonstrated early feasibility of using the UNICORN technique to prevent coronary obstruction during ViV-TAVR (Central Illustration). Further prospective trials are required to provide a more definitive assessment of the technique’s safety and efficacy.

### UNICORN technique

Coronary obstruction complicates approximately 2.3% of patients undergoing ViV-TAVR.^14^ Historically, chimney and snorkel stenting were the mainstay strategies for managing peri-TAVR coronary obstruction.^15^ More recently, the BASILICA technique has gained popularity,^16^^,^^17^ using transcatheter electrosurgery to lacerate the target leaflet and thereby reduce obstruction risk. The safety and efficacy of BASILICA have been demonstrated in both multicenter retrospective observational studies,^18^^,^^19^ and prospective investigational device exemption trials.^11^^,^^20^ In the latest European guideline for valvular heart disease, BASILICA is recommended as a leaflet modification strategy to prevent TAVR-related coronary obstruction.^21^

In 2022, we described UNICORN as an alternative leaflet modification strategy.^1^ There are 2 important distinctions between UNICORN and BASILICA. First, UNICORN uses mechanical force to open the target leaflet, rather than electrosurgical laceration.^17^ Second, when UNICORN is performed with BEV, the aim is to deploy the valve within an unruptured fenestration, minimizing aortic regurgitation time. Because the BEV remains in constant contact with the annulus, it shields the coronary ostium from prolapsing leaflets. Use of UNICORN and related techniques has expanded in recent years,^3^^,^^4^^,^^22^, ^23^, ^24^, ^25^, ^26^, ^27^, ^28^ although most published cases remain isolated case reports. Our registry addresses this gap by providing real-world data.

### Technical insights for early operators

Technical pearls for early operators include the following. First, as with BASILICA, precise leaflet traversal is a key procedural step. Traversal should target the leaflet base midway between 2 commissures, providing space for peripheral balloon dilatation and THV deployment, while maximizing coronary protection. Optimal projection angles for side and en face views, as determined by CT, are essential.^29^ In our experience, CT-derived angles for the LCC are usually feasible in the catheterization laboratory, whereas right coronary angles may occasionally be extreme and difficult to execute. In such cases, approximate fluoroscopic projections supplemented with real-time TEE guidance are critical to ensure accurate traversal. In addition to selecting the traversal site, the traversal trajectory and approach angle must be meticulously planned to achieve the correct wire entry direction and should be visualized and confirmed by TEE. Second, when BEV is used, the goal should be intraleaflet deployment, avoiding intentional upfront leaflet laceration. This approach minimizes the duration of aortic regurgitation and enhances coronary protection, as the BEV remains in constant contact with the annulus, shielding the coronary ostium from prolapsing leaflets. Third, although leaflet modification reduces the risk of coronary obstruction, we continue to recommend maintaining a low threshold for coronary protection if needed.

### Recrossing technique

A major goal of UNICORN with BEV is intraleaflet deployment. Unlike BEVs, SEVs lack the opening and radial force to lacerate the leaflet when expanded within a fenestration. Consequently, oversized balloons have been used to lacerate the leaflet before SEV deployment.^3^^,^^4^ This sequence creates a period of aortic regurgitation between leaflet laceration and valve implantation, which is usually tolerated but may be problematic in hemodynamically unstable patients. To address this, we recently described UNICORN with SEV using the recrossing technique.^5^ In this modification, the fenestration is recrossed from contralateral access with a second wire, which permits introduction of both a peripheral balloon and the SEV. The technique can be carried out in 2 ways. One approach is to lacerate the target leaflet with the peripheral balloon and then advance the SEV from the descending aorta for deployment. Alternatively, the peripheral balloon and SEV can be advanced together across the fenestration so that balloon inflation and valve release occur in immediate succession. By shortening the interval between leaflet laceration and valve implantation, both approaches reduce the period of aortic regurgitation and are especially valuable in patients with unstable hemodynamics.

The recrossing technique also serves as a bailout in BEV cases. If the BEV cannot be advanced through the fenestration, contralateral recrossing with a second wire and use of a larger peripheral balloon can further enlarge the opening, allowing successful valve deployment.

### Further development

Several areas warrant further study. First, unlike BASILICA, there is no prospective evidence for UNICORN; a multicenter trial is needed to establish safety and efficacy. Second, the optimal balloon size for fenestration dilatation likely depends on both the in situ and implanted valve types. Comprehensive systematic bench testing across various valve-balloon combinations, akin to the Valve-in-Valve App, is currently lacking.^30^ Further standardized bench testing is needed to guide procedural selection. It should be recognized that the actual valve behavior in vivo is influenced by the individual extent and mode of degeneration, which may not be fully represented in bench testing. Third, more experience is required with complex scenarios such as bileaflet modification with various combinational tactics.

### Study limitations

First, this study is limited by its single-center, retrospective design. Results from a single institution may be influenced by specific operator experience, institutional protocols, and patient selection, which may not reflect broader clinical practice. Therefore, multicenter prospective studies are needed to validate the safety, efficacy, and reproducibility of the technique across diverse settings. Second, not all combinations of in situ and implanted valves are represented. Systematic bench testing and broader clinical experience will be necessary to refine the technique and its generalizability. Third, the relatively small sample size limits the statistical power to identify the true effect of outcome variables and detect differences in comparator groups.

## Conclusions

The UNICORN Hong Kong Registry demonstrated early feasibility of using the UNICORN technique to prevent coronary obstruction during ViV-TAVR. Further prospective trials are required to provide a more definitive assessment of the technique’s safety and efficacy.

## Funding Support and Author Disclosures

This study received no funding. Dr Tang has received speaker honoraria and served as a physician proctor, consultant, advisory board member, TAVR publications committee member, RESTORE study steering committee member, APOLLO trial screening committee member, and IMPACT MR steering committee member for Medtronic; has received speaker honoraria and served as a physician proctor, consultant, advisory board member and TRILUMINATE trial anatomic eligibility and publications committee member for Abbott Structural Heart; has served as an advisory board member for Boston Scientific and JenaValve; a consultant and physician screening committee member for Shockwave Medical; a consultant for NeoChord, Peija Medical, and Shenqi Medical Technology; and has received speaker honoraria from Siemens Healthineers. All other authors have reported that they have no relationships relevant to the contents of this paper to disclose.
